# Assessment of the loading history of patients after high tibial osteotomy using SPECT/CT - a new diagnostic tool and algorithm

**DOI:** 10.1186/1749-799X-8-46

**Published:** 2013-12-10

**Authors:** Armin Mucha, Milos Dordevic, Enrique A Testa, Helmut Rasch, Michael T Hirschmann

**Affiliations:** 1 Department of Orthopaedic Surgery and Traumatology, Kantonsspital Baselland-Bruderholz, Bruderholz 4101, Switzerland; 2 Institute for Radiology and Nuclear Medicine, Kantonsspital Baselland-Bruderholz, Bruderholz 4101, Switzerland

**Keywords:** Knee, SPECT/CT, High tibial osteotomy, Diagnostics, Loading history, Pain, Realignment, Mechanical alignment

## Abstract

**Background:**

Single photon emission computerized tomography and conventional computerized tomography (SPECT/CT) tracer uptake has been shown to reflect the loading history of the tibiofemoral knee joint and correlate with the mechanical and anatomical alignment. It was our primary purpose to develop a novel standardized SPECT/CT algorithm for patients undergoing high tibial osteotomy, evaluate the inter- and intra-observer reliability (OR), and assess the clinical applicability for follow-up of patients before and after high tibial osteotomy.

**Methods:**

The localization scheme defines 9 femoral, 8 patellar, and 13 tibial zones to accurately map the examined tracer uptake volume in each anatomical area of interest. Maximum values for each area (mean ± standard deviation, median, and range) of the localization scheme were recorded as well as normalized values for the intensity of SPECT/CT tracer uptake calculated. The inter- and intra-OR was assessed for SPECT/CT localization and tracer activity. Pre- and postoperative mechanical alignment was assessed in SPECT/CT using a custom-made specialized software. The median inter- and intra-observer differences of the measured mechanical alignment were calculated along with the inter- and intra-OR.

**Results:**

The localization scheme showed near-perfect inter- and intra-OR (intra-class correlation coefficient (ICC) > 0.9) for the measurement of tracer activity and localization in all anatomical regions. For measurements of mechanical alignment, there was a strong agreement between the two observers (an inter-OR of ICC = 0.99 and an intra-OR of ICC = 0.98).

**Conclusions:**

The presented SPECT/CT algorithm is highly reliable and clinically feasible. Combined with mechanical alignment analysis, it provides the surgeon with helpful information about realignment effects of high tibial osteotomies (HTOs) and might help identify the optimal personalized degree of correction in HTO surgery.

## Background

Combined single photon emission computerized tomography and conventional computerized tomography (SPECT/CT) has yet proven useful as part of the diagnostic armamentarium in challenging orthopedic patients [1-7]. SPECT/CT tracer uptake is significantly correlated with the mechanical and anatomical alignment of the limb as well as with the degree of osteoarthritis [8]. It represents the loading history of the knee, and thus, overloading can be assessed at an early stage. Due to its unique characteristics, SPECT/CT promises information on the loading of the different compartments in the knee [8]. Thus, it offers great benefits for the assessment of patients after realignment procedures such as medial opening wedge high tibial osteotomy (OWHTO). Additionally, it allows an early detection of osteoarthritis.

Hirschmann et al. have shown how useful the combined assessment of alignment data and tracer uptake intensity distribution is in patients after total knee arthroplasty. Here, the remodeling of the bone with regard to the total knee arthroplasty position is assessed [4]. In patients after realignment surgery such as correction osteotomies, the deloading effect on the joint can be directly visualized.

However, neither a standardized algorithm for investigating patients after OWHTO nor a specific localization scheme to accurately map the examined tracer uptake volume in these patients has been published.

It was our primary purpose to introduce a novel standardized SPECT/CT algorithm including a specific localization scheme for patients undergoing high tibial osteotomy, evaluate the inter- and intra-observer reliability, and assess the clinical applicability for follow-up of patients after high tibial osteotomy. The proposed algorithm aims to improve the evaluation of patients before and after high tibial osteotomy.

## Methods

Twenty-two consecutive patients (male/female 17:5, mean age 47 ± 10) with 23 knees (male/female 18:5) undergoing medial OWHTO for medial joint compartment overloading or medial tibiofemoral osteoarthritis due to mechanical varus alignment were prospectively included in this study. Exclusion criteria were posttraumatic osteoarthritis, open growth plates, corticosteroid use within the last 6 months, known history of avascular necrosis, osteochondritis dissecans, meniscal chondrocalcinosis, tumor, Paget's disease, joint infection, periarticular fracture, neuropathic arthropathy, reactive arthritis, gout, or a pregnancy.

^99m^Tc-hydroxymethylene-diphosphonate (HDP)-SPECT/CT images of these consecutive patients were prospectively collected and then evaluated. The study was approved by the local ethical committee. All procedures were performed in accordance with the ethical standards of the responsible committee and with the guidelines of the Helsinki Declaration of 1975, as revised in 2008. All patients gave written informed consent before inclusion into the study.

SPECT/CT was performed using a hybrid system (Symbia T16, Siemens, Erlangen, Germany), which is equipped with a pair of low-energy, high-resolution collimators and a dual-head gamma camera with an integrated 16-slice CT scanner (collimation of 16 × 0.75 mm). In all patients, a 500-MBq ^99m^Tc-HDP tracer (Mallinckrodt, Wollerau, Switzerland) was used.

Planar scintigraphic images were taken in three phases: the perfusion phase (immediately after injection), the soft tissue phase (from 1 to 5 min after injection), and the delayed metabolic phase (from 2 h after injection). SPECT/CT was performed with a matrix size of 128 × 128, an angle step of 32, and a time per frame of 25 s 2 h after injection. The CT protocol was modified according to the Imperial Knee Protocol [9].

Reconstructed data was displayed in axial, coronal, and sagittal planes. SPECT/CT images were analyzed using a customized validated software (IntroSPECT©, OrthoImagingSolutions Ltd., London, UK), which is able to quantify tracer uptake volumetrically in 3D and anatomically localize these areas of increased tracer uptake [10].

### Specific tracer uptake analysis

**Figure 1:**
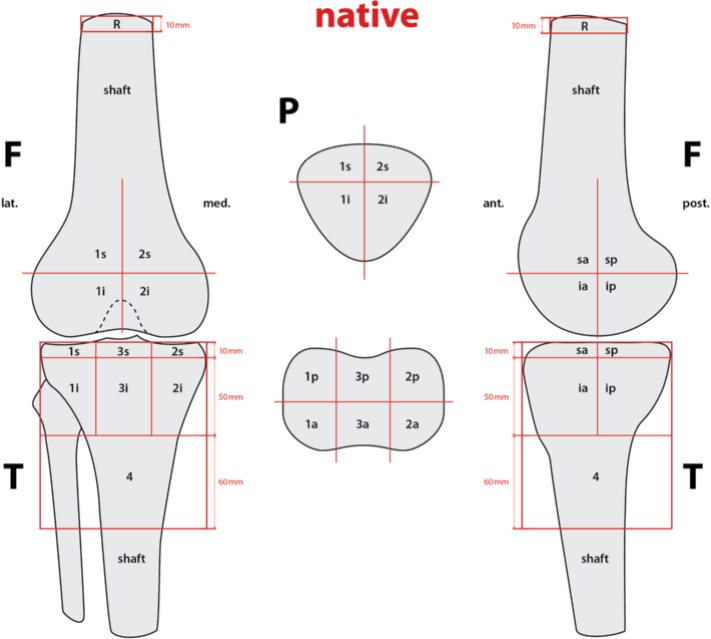
**Mapping scheme used for localization of areas of increased SPECT/CT tracer uptake values in native knees.***F* femur, *T* tibia, *P* patella, *R* reference zone, *1* medial, *2* lateral, *3* tibial intercondylar area, *4* distal plate area, *s* superior, *i* inferior, *a* anterior, *p* posterior.

**Figure 2:**
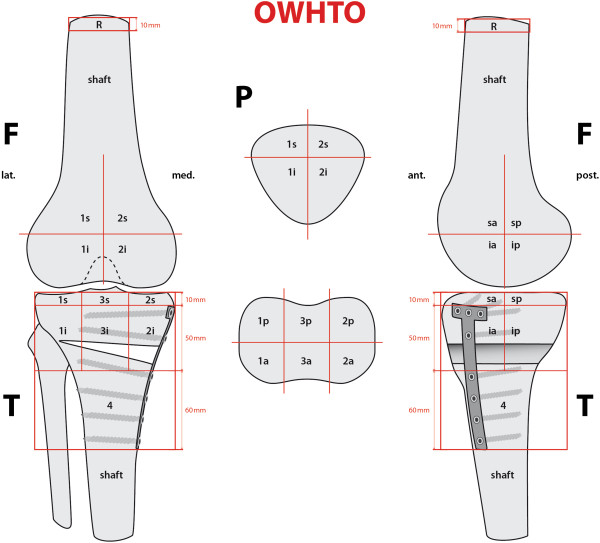
**Mapping scheme used for localization of areas of increased SPECT/CT tracer uptake values in knees after OWHTO.***F* femur, *T* tibia, *P* patella, *R* reference zone, *1* medial, *2* lateral, *3* tibial intercondylar area, *4* distal plate area, *s* superior, *i* inferior, *a* anterior, *p* posterior.

The proposed localization scheme defines 9 femoral, 8 patellar, and 13 tibial zones to accurately map the examined tracer uptake volume in each anatomical area of interest. In order to have comparable volumes, we used an identical localization scheme adjusted to both native (Figure 1) and operated (Figure 2) knees. Maximum values (mean ± standard deviation, median, and range) for each area of the localization scheme were recorded as well as normalized values for the intensity of SPECT/CT tracer uptake calculated. For normalization, a specific area within the femoral shaft was used as the reference region, as previously published [10]. Thus, obtained values were dimensionless and comparable.

**Figure 3:**
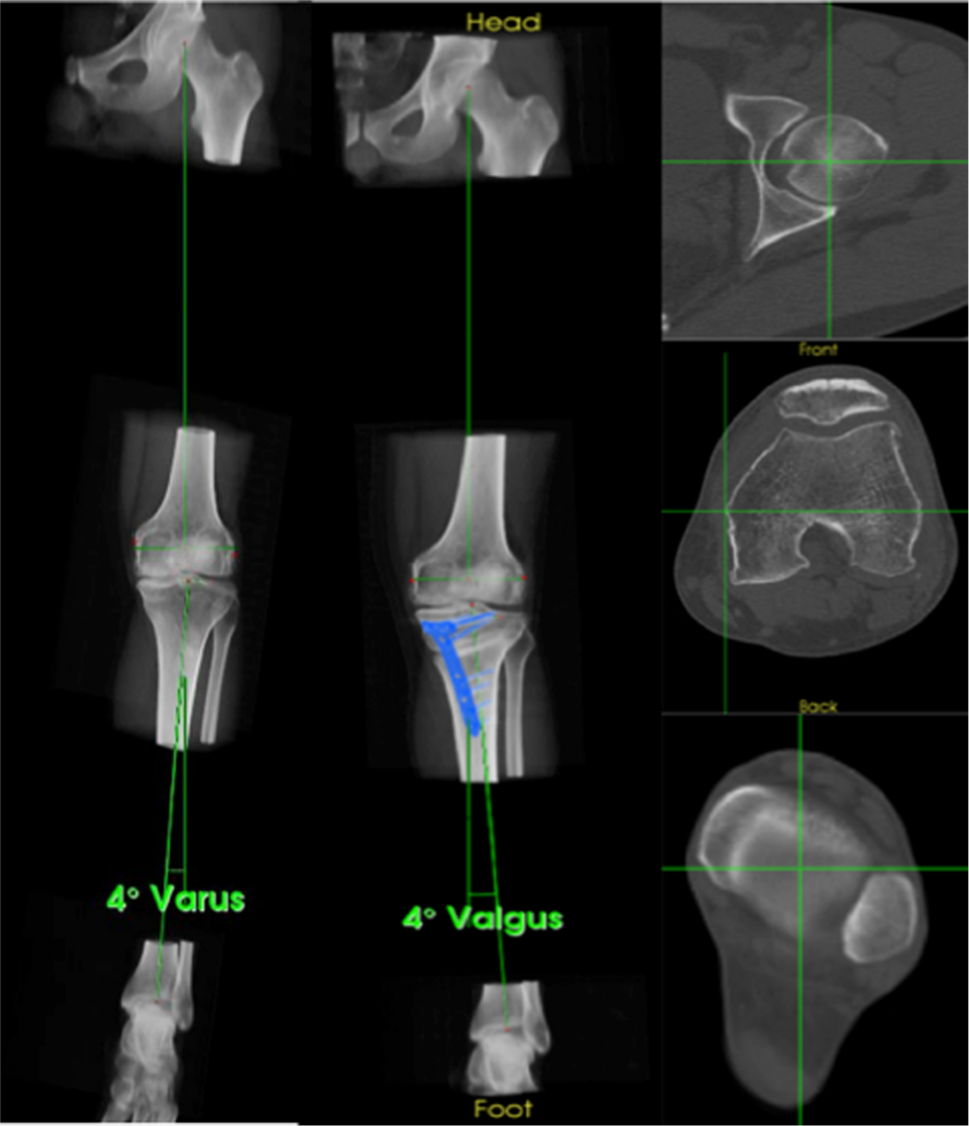
**Pre- and postoperative mechanical alignment on SPECT/CT.** The lower limb is aligned using the mechanical axis determined by the femoral head center, the medial and lateral femoral epicondyles, the center of the tibial plateau, and the talar center. Here, a pre- (*4° Varus*) and a postoperative (*4° Valgus*) knee are shown.

Pre- and postoperative mechanical alignment on SPECT/CT is explained in Figure 3. It was assessed on SPECT/CT using a custom-made previously validated software (Orthoexpert v1.15©, OrthoImagingSolutions Ltd., London, UK) [11].

All measurements were done twice by two independent observers with a 2-week interval between analyses. Both observers were blinded to results from previous observations.

### Statistical analysis

Data were analyzed using SPSS 17.0 (SPSS, Chicago, IL, USA). Sample size was calculated according to the reported estimates for reliability studies using intra-class correlation coefficients (ICCs) [12].

The inter- and intra-observer reliability of the localization scheme, grading of the tracer activity, and mechanical alignment measurements were determined by calculating the ICC. An ICC value of 1 indicates perfect reliability, 0.81–1 very good reliability, and 0.61–0.80 good reliability [12]. The inter- and intra-observer variabilities in mechanical alignment measurements were also calculated.

## Results

**Figure 4:**
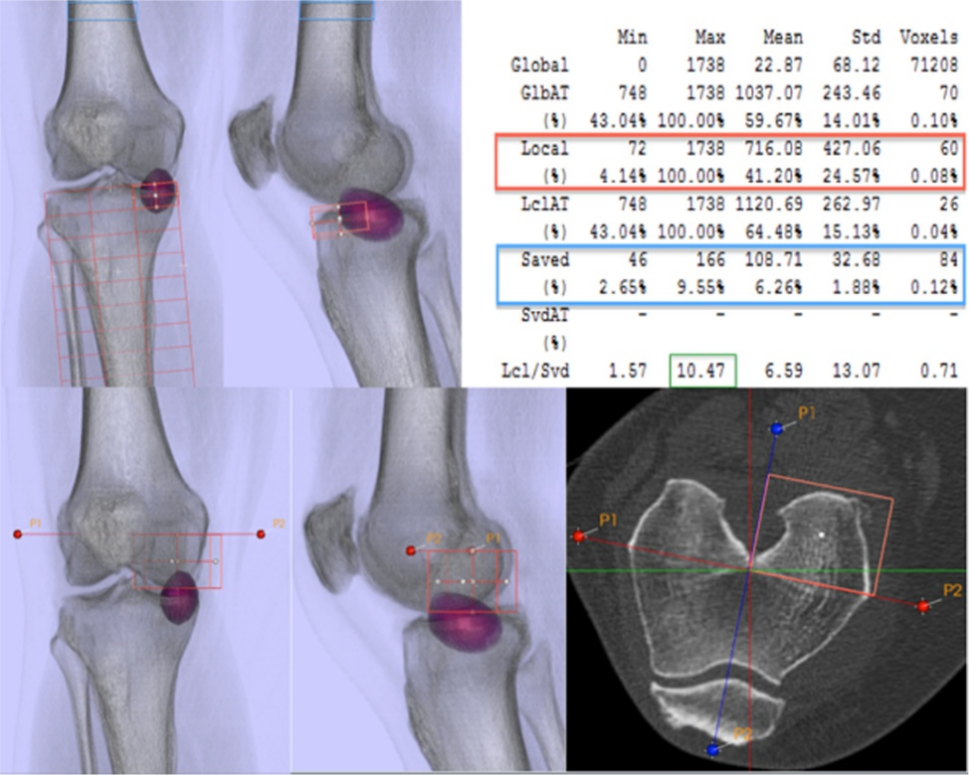
**Localization scheme application on a native knee of a 54-year-old female patient showing medial overloading.** Normalized value (*green*) of maximum SPECT/CT tracer uptake in a defined zone is obtained by dividing the volume of interest (*orange*) by the reference region (*blue*)*.*

The localization scheme proved to be useful and easily applicable in all 23 knees. The scheme defined the relevant areas, and the ^99m^Tc-HDP tracer uptake could be located to specific anatomical regions in all cases. Mean ICC for inter- and intra-observer reliability (OR) for all regions (femur, tibia, and patella) and both schemes ranked around 0.99 and 0.97, respectively. Inter- and intra-OR for pre- and postoperative adjusted schemes were calculated separately, showing high ICCs for both schemes. The ICCs are presented in Tables 1 and 2. Figure 4 shows the application of the localization scheme in a typical patient.

**Table 1:** **Inter- and intra-OR (ICC) of **^**99m**^**Tc-HDP-SPECT/CT activity using the preoperative localization scheme for all regions**

**Location**	**Type**	**Intra-observer reliability**		**Inter-observer reliability**
		**Observer 1**	**Observer 2**	**Observer 1-Observer 2**
F1sa	Ratio	0.995	0.958	0.988
F2sa	Ratio	0.959	0.992	0.989
F1sp	Ratio	1	1	1
F2sp	Ratio	0.999	0.999	1
F1ia	Ratio	0.985	0.953	0.988
F2ia	Ratio	0.983	0.97	0.988
F1ip	Ratio	0.989	0.983	0.999
F2ip	Ratio	0.997	0.994	0.998
P1s	Ratio	0.996	0.996	0.998
P2s	Ratio	0.996	0.986	0.996
P1i	Ratio	0.944	0.985	0.982
P2i	Ratio	0.907	0.942	0.985
T1sa	Ratio	0.981	0.984	0.997
T3sa	Ratio	0.993	0.99	0.998
T2sa	Ratio	0.947	0.975	0.984
T1sp	Ratio	0.993	0.987	0.997
T3sp	Ratio	0.977	0.98	0.989
T2sp	Ratio	0.993	0.991	0.996
T1ia	Ratio	0.984	0.964	0.99
T3ia	Ratio	0.982	0.97	0.989
T2ia	Ratio	0.898	0.975	0.977
T1ip	Ratio	0.997	0.937	0.985
T3ip	Ratio	0.982	0.98	0.988
T2ip	Ratio	0.975	0.994	0.993
T4	Ratio	0.969	0.999	0.993
Total	Ratio	0.977	0.979	0.991

**Table 2:** **Inter- and intra-OR (ICC) of **^**99m**^**Tc-HPD-SPECT/CT activity using the postoperative localization scheme for all regions**

**Location**	**Type**	**Intra-observer reliability**		**Inter-observer reliability**
		**Observer 1**	**Observer 2**	**Observer 1-Observer 2**
F1sa	Ratio	0.991	0.976	0.97
F2sa	Ratio	0.96	0.97	0.999
F1sp	Ratio	0.94	0.999	0.994
F2sp	Ratio	0.779	0.988	0.986
F1ia	Ratio	0.993	0.992	0.982
F2ia	Ratio	0.979	0.92	0.948
F1ip	Ratio	0.983	0.975	0.996
F2ip	Ratio	0.958	0.965	0.974
P1s	Ratio	0.997	0.987	0.991
P2s	Ratio	0.965	0.976	0.983
P1i	Ratio	0.971	0.989	0.991
P2i	Ratio	0.974	0.983	0.987
T1sa	Ratio	0.958	0.925	0.979
T3sa	Ratio	0.979	0.989	0.965
T2sa	Ratio	0.972	0.963	0.984
T1sp	Ratio	0.973	0.983	0.992
T3sp	Ratio	0.997	0.992	0.99
T2sp	Ratio	0.956	0.986	0.995
T1ia	Ratio	0.983	0.985	0.994
T3ia	Ratio	0.988	0.987	0.983
T2ia	Ratio	0.963	0.929	0.997
T1ip	Ratio	0.995	0.998	0.994
T3ip	Ratio	0.983	0.996	0.968
T2ip	Ratio	0.999	0.736	0.999
T4	Ratio	0.998	0.992	0.999
Total	Ratio	0.969	0.967	0.933

**Figure 5:**
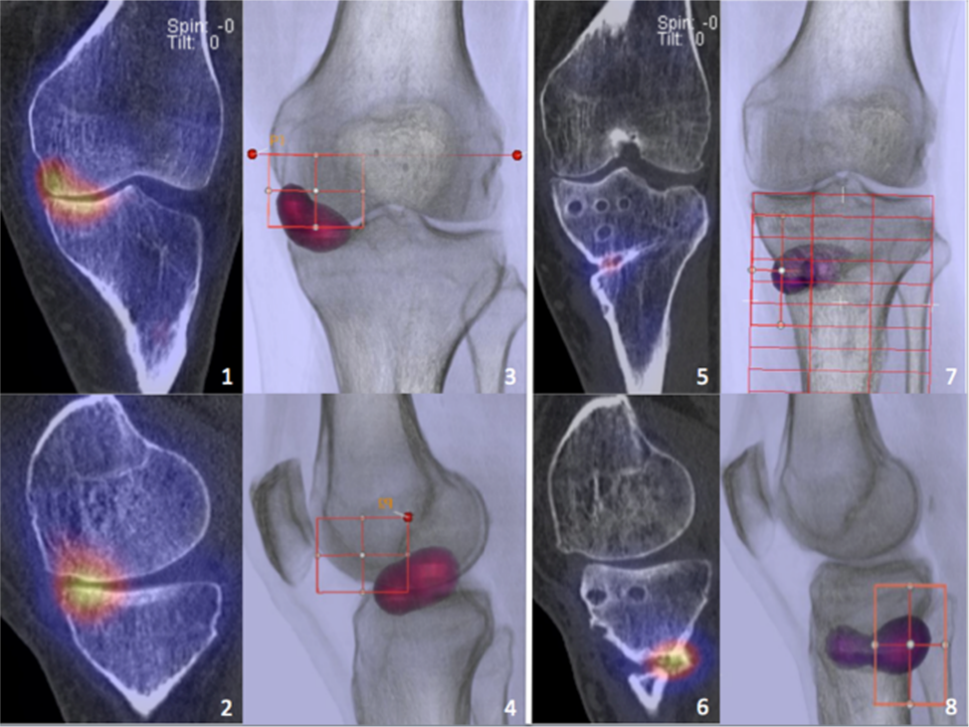
**SPECT/CT images and 3D reconstructions before and after OWHTO.***Left:* SPECT/CT images (*1*, *2*) and 3D radiolucent reconstructions (*3*, *4*) of a 55-year-old patient before OWHTO showing medial compartment overloading. *Right:* 1-year postoperative SPECT/CT images (*5*, *6*) and 3D reconstructions (*7*, *8*) reveal an unloading effect of the surgically achieved correction and an advancing consolidation of the osteotomy gap with physiological increased osseous metabolism.

SPECT/CT images were of sufficient quality to determine the necessary anatomical landmarks (Figure 5). When comparing the obtained measurements for alignment, a strong agreement between the two observers was found: overall mean ICCs were 0.99 (inter-OR) and 0.98 (intra-OR). The median differences (ranges) in mechanical alignment measurements before and after osteotomy between the two observers (inter-observer) and within the measurements of the first observer (intra-observer) are shown in Table 3.

**Table 3:** Median differences (ranges) of pre- and postoperative mechanical alignment for intra- and inter-observer testing

	**Preoperative**	**Postoperative**
Inter-observer variation (deg)	1 (0–2)	1 (0–2)
Intra-observer variation (deg)	1 (0–1)	0 (0–2)

## Discussion

Despite the paucity of yet available literature on the use of SPECT/CT in orthopedic patients, several promising applications of this imaging modality have been reported [2,4,6,8,10,13,14]. The purpose of this study was to evaluate the applicability of a SPECT/CT localization scheme and its potential benefits in patients before and after HTO.

The most important findings of this study were as follows: The proposed algorithm for investigating patients before and after OWHTO using a specific localization scheme to accurately allocate the SPECT/CT tracer uptake proved to be clinically feasible in all patients showing near-perfect inter- and intra-OR. This is the first study to describe SPECT/CT tracer uptake in knees after HTO.

The described method of assessment of the mechanical leg axis is easily applicable and also showed high inter- and intra-OR. Well-defined and reliable bony landmarks such as center of the femoral head, anatomical epicondylar line, center of the tibial plateau, and talar center were used as reference points for determination of alignment [9,15,16].

For effective unloading of the medial knee compartment, Fujisawa et al. [17] recommended to correct the weight bearing line to a point 62% laterally on the tibial plateau diameter. To date, the degree of alignment correction by HTO is mainly based on the findings of Fujisawa et al. with only minor adjustments in recent years [18]. However, the question of which optimal correction should be achieved in every patient remains unanswered. Due to its unique visualization of the HTO unloading effect, the proposed algorithm might be helpful in establishing an individualized degree of correction and deciding whether the operated knee was adequately, over-, or undercorrected. Clearly, it can be expected that a preoperatively increased bone tracer uptake in the medial compartment will be reduced after a well-performed HTO, a finding which can be confirmed from our data here. In addition, we have proven in a previous study that bone tracer uptake in SPECT/CT reflects the loading pattern of the knee joint [8].

Since diphosphonate-based tracer (^99m^Tc-HDP and ^99m^Tc-MDP) uptake is known to correlate with osteoarthritic joint changes and with knee overloading [8,19,20], SPECT/CT can be considered as a valuable diagnostic tool for an early detection of patients who considerably benefit from a timely realignment treatment such as HTO.

Due to the present study, SPECT/CT should be used for follow-up of patients before and after realignment surgery such as high tibial osteotomy or patellofemoral deloading.

However, the use of SPECT/CT should still be carefully indicated as the radiation burden to the patient equals three to four times that of a long leg radiograph. Aiming for a further reduction of the radiation dose, we introduced a specific low-dose SPECT/CT protocol [9]. Moreover, SPECT/CT may substitute lengthy series of less conclusive imaging modalities such as long leg radiographs and thereby achieve a reduction of the overall radiation burden.

Current SPECT image resolution is still considerably lower (4 mm) than CT (0.7 mm). This mismatch does lead to some inaccuracy of SPECT localization. However, in future SPECT/CT systems, this will be resolved.

## Conclusions

In summary, the presented SPECT/CT algorithm is highly reliable and clinically feasible. Evaluation of patients before and after OWHTO using SPECT/CT with regard to the mechanical leg alignment provides the surgeon with helpful additional information about the loading history of the knee joint. The clinical utility of this proposed algorithm and its localization scheme should be further investigated in clinical studies.

## Competing interests

The authors declare that they have no competing interests.

## Authors’ contributions

AM, MD, ET, HR, and MTH participated in the design of the study, performed the data analysis, and drafted the manuscript. All authors read and approved the final manuscript.
